# Crosstalk between p38 and Erk 1/2 in Downregulation of FGF1-Induced Signaling

**DOI:** 10.3390/ijms20081826

**Published:** 2019-04-12

**Authors:** Malgorzata Zakrzewska, Lukasz Opalinski, Ellen M. Haugsten, Jacek Otlewski, Antoni Wiedlocha

**Affiliations:** 1 Department of Protein Engineering, Faculty of Biotechnology, University of Wroclaw, 50-383 Wroclaw, Poland; lukasz.opalinski@uwr.edu.pl (L.O.); jacek.otlewski@uwr.edu.pl (J.O.); 2 Department of Tumor Biology, Institute for Cancer Research, Oslo University Hospital, Montebello, 0379 Oslo, Norway; ellen.m.haugsten@rr-research.no; 3 Centre for Cancer Cell Reprogramming, Institute of Clinical Medicine, Faculty of Medicine, University of Oslo, Montebello, 0379 Oslo, Norway; antoni.wiedlocha@rr-research.no; 4 Department of Molecular Cell Biology, Institute for Cancer Research, Oslo University Hospital, Montebello, 0379 Oslo, Norway

**Keywords:** FGF-induced signaling, FRS2, phosphorylation, downregulation, p38, MAPK

## Abstract

Mitogen-activated protein kinases (MAPK): Erk1 and Erk2 are key players in negative-feedback regulation of fibroblast growth factor (FGF) signaling. Upon activation, Erk1 and Erk2 directly phosphorylate FGF receptor 1 (FGFR1) at a specific serine residue in the C-terminal part of the receptor, substantially reducing the tyrosine phosphorylation in the receptor kinase domain and its signaling. Similarly, active Erks can also phosphorylate multiple threonine residues in the docking protein FGF receptor substrate 2 (FRS2), a major mediator of FGFR signaling. Here, we demonstrate that in NIH3T3 mouse fibroblasts and human osteosarcoma U2OS cells stably expressing FGFR1, in addition to Erk1 and Erk2, p38 kinase is able to phosphorylate FRS2. Simultaneous inhibition of Erk1/2 and p38 kinase led to a significant change in the phosphorylation pattern of FRS2 that in turn resulted in prolonged tyrosine phosphorylation of FGFR1 and FRS2 and in sustained signaling, as compared to the selective inhibition of Erks. Furthermore, excessive activation of p38 with anisomycin partially compensated the lack of Erks activity. These experiments reveal a novel crosstalk between p38 and Erk1/2 in downregulation of FGF-induced signaling.

## 1. Introduction

Fibroblast growth factor (FGF) receptor (FGFR) -dependent signaling plays a crucial role during embryonic development, as well as in adult life. It stimulates growth, differentiation, survival, injury repair, regeneration, and metabolism. Consequently, excessive activation of FGFRs may result in severe abnormalities, such as cancer development and progression, and skeletal disorders [1].

Downregulation of FGFR signaling consists of a number of mechanisms that enable precise control of cellular outcome. They include regulation of FGFR synthesis, receptor internalization, followed by degradation and modulation of FGFR tyrosine phosphorylation via the activity of several negative regulators and feedbacks [1,2,3,4,5,6].

Previously we showed that phosphorylation of Ser777 of FGFR1 by activated MAPK Erk1 and Erk2 significantly reduces the tyrosine phosphorylation in the kinase domain of the receptor. We suggested that the phosphorylated Ser777 could act as a binding site for tyrosine phosphatases responsible for receptor inactivation [7]. FGFR1 and FGFR2 are also phosphorylated on Ser779 in response to FGF. Upon phosphorylation, this residue serves as a binding site for 14-3-3 family of phosphoserine/threonine-binding adaptor/scaffold proteins and tunes up Ras/MAPK signaling [8,9]. In addition, ribosomal S6 kinase 2 (RSK2) phosphorylates Ser789 of FGFR1. Inhibition of RSK2 activity leads to prolonged tyrosine phosphorylation of FGFR1 resulting from reduced FGFR1 ubiquitination and endocytosis [10].

Furthermore, FGFR downstream signaling molecules are also affected by inhibitory loops and negative regulators, including Sprouty proteins (SPRY1–SPRY4), MAPK phosphatase 3 (MKP3), SEF (similar expression to FGF), and protein tyrosine phosphatase receptor type G (PTPRG). MKP3 directly dephosphorylates MAPK turning off the Ras cascade [11]. Sprouty is phosphorylated in response to FGFR activation and competes for Grb2 (growth factor receptor-bound protein 2), binding to FRS2 and Shp2 (SH2 domain-containing protein tyrosine phosphatase-2), preventing Ras activation. Phosphorylated Sprouty also binds to Raf and blocks subsequent MAPK signaling [12,13]. SEF protein, in addition to its role in inhibiting MAPK signaling, is capable of interacting directly with FGFRs and blocking receptor phosphorylation [14,15]. The protein tyrosine phosphatase receptor type G, PTPRG, directly dephosphorylates the receptor itself, thereby turning off the signaling [16].

However, one of the most effective downregulation mechanism described so far is based on Erk1/2-FRS2 negative feedback loop [17]. FRS2, a lipid anchored adaptor protein, is associated constitutively through its PTB domain with the juxtamembrane domain of FGFR and is phosphorylated on multiple tyrosines upon receptor activation [18,19]. Phosphorylated FRS2 forms two specific binding sites for Shp2 and four binding sites for Grb2. Grb2 is constitutively bound via its SH3 domains to Sos and Gab1, and these proteins constitute a signaling complex activating the RAS/MAPK and PI3K/Akt pathways [1]. Activated extracellular signal-regulated protein kinases (Erk1/2), irrespective of the upstream receptor tyrosine kinase (RTK), phosphorylate FRS2 at eight threonines (PXTP motifs) [17]. Such phosphorylation reduces FRS2 tyrosine phosphorylation, decreases recruitment of Grb2, and attenuates downstream signaling response, providing a control mechanism to regulate FGFR activity [17].

Besides Erks, the MAPK family includes also p38 and c-Jun N-terminal kinases (JNKs). Erks are activated by mitogenic factors and are associated with growth, differentiation, and proliferation, whereas p38 and JNKs take part mainly in the cell response to stress conditions and inflammatory cytokines, and are activated only weakly by proliferative stimuli [20]. There are four isoforms of p38 identified so far in mammals, among which p38α and p38β are the best characterized and most widely expressed [21].

Interestingly, all MAPKs share specificity for a common consensus phosphorylation site [22]. We showed previously that two different MAPKs, Erk1/2 and p38, are able to phosphorylate FGFR1 at the same serine residue [7,23]. Phosphorylation of FGFR1 by p38 seems to be necessary for FGF1 to be translocated into the cytosol and nucleus upon internalization [23], while phosphorylation by Erk1/2 provides a negative-feedback mechanism that controls FGF signaling, and thereby protects the cell against excessive activation of FGFR [7].

Here, we found that in addition to Erks, p38 can also phosphorylate FRS2 and modulate its phosphorylation at tyrosine residues. Simultaneous inhibition of both types of MAPKs resulted in prolonged activation of FGFR1, FRS2, and downstream signaling, as compared to the elimination of Erks activity alone. Our results reveal a novel regulatory feedback mechanism, where p38 is able to partially substitute Erk1 and Erk2 in the regulation of FGFR activity.

## 2. Results

### 2.1. The p38 Activity Influences Electrophoretic Mobility Shift of FRS2

**Figure 1:**
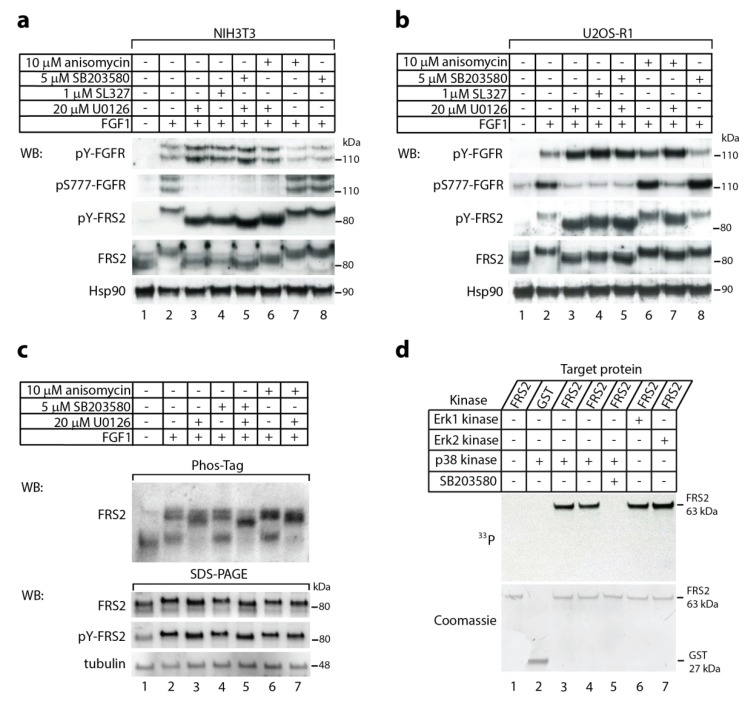
The effect of p38 activity on FRS2. (**a**–**c**) FGF1-induced electrophoretic mobility shift of FRS2. Serum-starved (**a**,**c**) NIH3T3 and (**b**) U2OS-R1 were pretreated for 20 min with or without MEK1/2 inhibitors (20 µM U0126, 1 µM SL327), p38 inhibitor (5 µM SB203580), and p38 activator (10 µM anisomycin), and then stimulated with the growth factor in the presence of heparin (10 U/mL) for 15 min. Cells were lysed, and the cellular material was analyzed by sodium dodecyl sulfate polyacrylamide gel electrophoresis (SDS-PAGE) (**a**,**b**) or Phos-Tag SDS-PAGE (**c**) and immunoblotting using the following antibodies: anti-phospho-FGFR (Tyr653/Tyr654) (pY-FGFR), anti-phospho-FGFR1 (Ser777) (pS777-FGFR1), anti-phospho-FRS2 (Tyr196) (pY-FRS), anti-FRS2, and anti-Hsp90 or anti-tubulin as a loading control. (**d**) In vitro phosphorylation of FRS2 by p38α kinase. Recombinant, active kinase p38α and partial recombinant fusion protein FRS2α with GST tag were incubated with (γ-^33^P) ATP in reaction buffer at 30 °C for 30 min in the presence or absence of 5 µM SB203580. Erk1 and Erk2 kinases served as positive controls. The proteins were analyzed by SDS-PAGE, electroblotting, and autoradiography (upper panel), and then the membrane was stained with Coomassie (lower panel). Representative experiments are shown, for (**a**) and (**b**) n = 4, and for (**c**) and (**d**) n = 2.

We analyzed by immunoblotting the activation of FGF1-induced signaling in NIH3T3 mouse fibroblast cells upon 15-min stimulation with FGF1 in the presence of different combinations of MAP kinases inhibitors (MEK1/2 inhibitors, p38 inhibitor) and p38 kinase activator (anisomycin) (Figure 1a). As expected, in the presence of specific MEK1/2 inhibitors (U0126 or SL327, lanes 3, 4, 5, 6) that prevent Erk1/2 activation, we observed augmented tyrosine phosphorylation of FGFR1 (Tyr653/Tyr654) and lack of phosphorylation at Ser777. In addition, MEK1/2 inhibition led to increased tyrosine phosphorylation of FRS2. This is in accordance with findings by Lax and co-workers [17]. The band for FRS2, visualized by antibodies recognizing total and phoshorylated (Tyr196) forms, displayed faster migration (lanes 3 and 4) as compared to the cells untreated with inhibitor (lane 2). Interestingly, we also found that in the simultaneous presence of MEK1/2 inhibitor (U0126) and p38 inhibitor (SB203580) (lane 5), the FRS2 band migrated slightly faster than in the presence of U0126 or SL327 alone (lanes 3 and 4). Addition of anisomycin (p38 activator) in the presence of U0126 resulted in a small up-shift of the FRS2 band (lane 6). We confirmed these findings using another model cell line, human osteosarcoma U2OS cells that stably expresses FGFR1 (U2OS-R1) (Figure 1b).

As conventional SDS-PAGE is not optimal for separation of phosphorylated populations of a protein from a non-modified fraction, we studied FRS2 migration upon treatment with various kinases inhibitors using a Phos-Tag technology that is well suited for phosphorylation profiling of proteins [24,25]. Cell lysates were separated on Phos-Tag gels followed by immunoblotting with anti-FRS2 antibody (Figure 1c).

In the serum starved NIH3T3 cells, FRS2 was observed mainly as a single, fast migrating band on Phos-Tag gel, likely representing non-phosphorylated FRS2 (Figure 1c, lane 1). Stimulation of cells with FGF1 drastically changed the migration pattern of FRS2 in the Phos-Tag gel. FRS2 was detected in three slowly migrating bands that represent differentially phosphorylated FRS2 (Figure 1c, lane 2). Treatment of cells with MEK1/2 inhibitor caused clear changes in the phosphorylation status of FRS2 (Figure 1c, lane 3). Interestingly, concomitant inhibition of p38 and MEK1/2 altered gel migration of FRS2 in relation to the inhibition of either MEK1/2 or p38 alone (Figure 1c, lane 5 vs. lanes 3 and 4). In agreement with these findings, the over-activation of p38 by anisomycin, either in the presence or absence of MEK1/2 inhibitor, altered the migration of FRS2 on Phos-Tag gels (Figure 1c, lanes 6 and 7 vs lanes 2 and 3). These data suggest that p38 kinase affects the phosphorylation state of FRS2.

### 2.2. In Vitro Phosphorylation of FRS2 by p38α Kinase

Since the addition of either p38 inhibitor or p38 activator resulted in FRS2 mobility changes in SDS-PAGE and Phos-Tag PAGE, we hypothesized that p38, in addition to Erks, is also able to phosphorylate FRS2. To verify such possibility, we performed in vitro phosphorylation using recombinant FRS2 and recombinant active form of p38α kinase. Using autoradiography we observed a clear band corresponding to phosphorylated FRS2 when active p38α was present during the reaction (Figure 1d, lanes 3 and 4, two parallel samples). FRS2 phosphorylation was dependent on p38 activity, since in the presence of the specific p38 inhibitor, SB203580, there was no trace of radioactivity (Figure 1d, lane 5). Interestingly, we did not observe any differences in the efficiency of phosphorylation (assessed by intensity of bands) of FRS2 in the case of p38α and both Erks (Erk1/2) (Figure 1d). These data indicate that p38 can phosphorylate FRS2.

### 2.3. Synergistic Effect of MEK1/2 and p38 Inhibitors on Kinetics of FGF1-Induced Signaling

**Figure 2:**
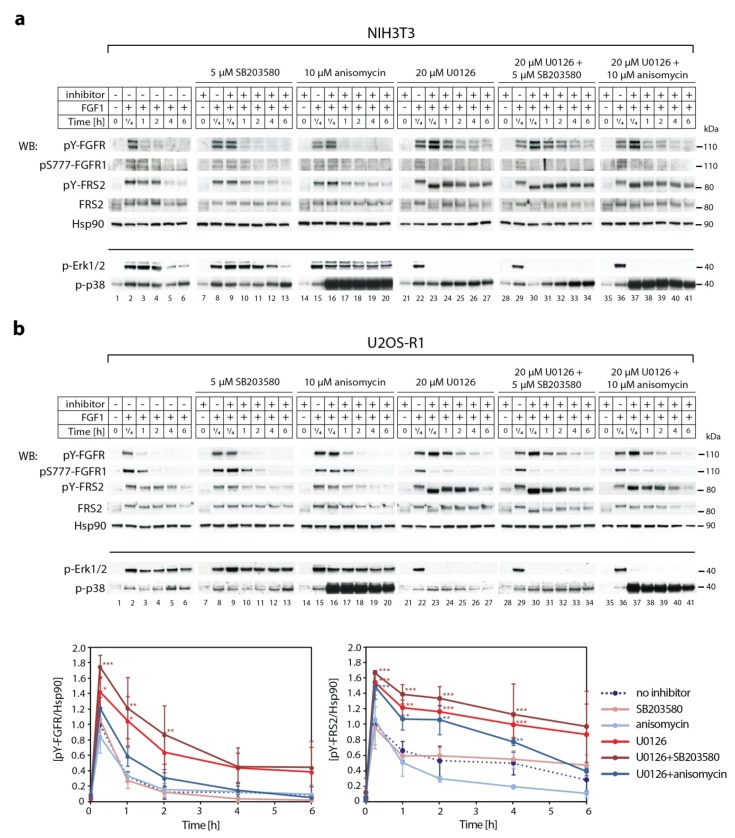
The crosstalk between p38 and Erk1/2 in downregulation of FGF1-induced signaling. Serum-starved (**a**) NIH3T3 and (**b**) U2OS-R1 cells were pretreated for 30 min with or without 20 µM U0126, 5 µM SB203580 and 10 µM anisomycin, and then stimulated with the growth factor in the presence of heparin (10 U/mL) and brefeldin A (2 μg/mL) for different time points. Cells were lysed, and the cellular material was analyzed by SDS-PAGE and immunoblotting using the following antibodies: anti-phospho-FGFR (Tyr653/Tyr654) (pY-FGFR), anti-phospho-FGFR1 (Ser777) (pS777-FGFR1), anti-phospho-FRS2 (Tyr196) (pY-FRS), anti-FRS2, and anti-Hsp90 as a loading control. Anti-phospho-Erk1/2 (p-Erk1/2) and anti-phospho-p38 MAPK (Thr180/Tyr182) (p-p38) were used to control the effect of U0126 (MEK inhibitor) and anisomycin (p38 activator). Representative experiments are shown, n = 3. The graphs present quantification of bands from panel b corresponding to phospho-FGFR1 (Tyr653/Tyr654) and phospho-FRS2 (Tyr196) normalized to loading control (Hsp90) and expressed as a fraction of the maximal response in the absence of inhibitor. Data are means ± SD of three independent experiments; * *p* < 0.05, ** *p* < 0.01, *** *p* < 0.001.

Next, we studied the impact of p38 activity on the kinetics of signaling cascades activated by FGF1 in NIH3T3 (Figure 2a). Experiments were performed in the presence of brefeldin A (2 μg/mL) to prevent the appearance of newly synthesized receptors [7]. In the presence of 5 µM of SB203580, p38 inhibitor (Figure 2a, lanes 9–13), or 10 µM anisomycin, p38 activator (Figure 2a, lanes 16–20), we did not observe significant differences in the intensity of FRS2 phosphorylation and FRS2 mobility, as compared to untreated cells. However, when the cells were simultaneously treated with 20 µM of MEK1/2 inhibitor (U0126) and either p38 inhibitor (SB203580) or p38 activator (anisomycin), the phosphorylation pattern of FRS2 varied, as well as the duration of FGFR1 tyrosine phosphorylation. We observed a synergistic effect of SB203580 and U0126. When activity of both kinds of MAP kinases (Erks and p38) were blocked, not only was the intensity of the band corresponding to tyrosine-phoshorylated FGFR1 stronger and lasted longer (Figure 2a, lanes 30–34), but also the electrophoretic mobility shift of FRS2 was prolonged, as compared to the inhibition of MEK1/2 with U0126 alone (Figure 2a, lanes 23–27). Fifteen minutes after FGF1 stimulation, FRS2 migrated faster in the presence of U0126, as well as in the presence of U0126 and SB203580, than in the absence of inhibitors. In the course of the experiment, the FRS2 band shifted upwards. When Erks and p38 were blocked at the same time (Figure 2a, lanes 30–34), we observed a more gradual change in the position of the FRS2 band than in the presence of U0126 alone (Figure 2a, lanes 23–27). In contrast to the effect of p38 inhibition, hyper-activation of p38 with 10 µM anisomycin used in the combination with 20 µM U0126 compensated, to some extent, the lack of Erk activity (Figure 2a, lanes 37–41). In the presence of anisomycin and U0126, the FRS2 band was less down-shifted after 15-min treatment with FGF1 and moved to the direction of higher molecular masses much faster over time than in the case of cells treated only with U0126. We confirmed all these findings in U2OS-R1 cells (Figure 2b). These data demonstrate that Erks and p38 regulate phosphorylation status of FRS2, which in turn modulates kinetics of FGFR1 signaling (Figure 2b).

### 2.4. The Effect of p38 Kinase on Erk1/2 Activity

Finding a synergistic effect of the inhibition of both types of MAP kinases, Erks and p38, we asked if they were able to compensate for each other. Indeed, we observed that FGF1-dependent Erk1/2 phosphorylation in the presence of p38 inhibitor was slightly stronger (Figure 2a, lanes 8 and 9), whereas in the presence of p38 activator (anisomycin), was notably weaker (Figure 2a, lanes 15 and 16) as compared to the untreated cells (Figure 1a, lane 2). Analysis of signaling kinetics revealed that the inhibition of Erks increased the phosphorylation of p38 (Figure 2a, lanes 23–27 and lanes 30–34).

**Figure 3:**
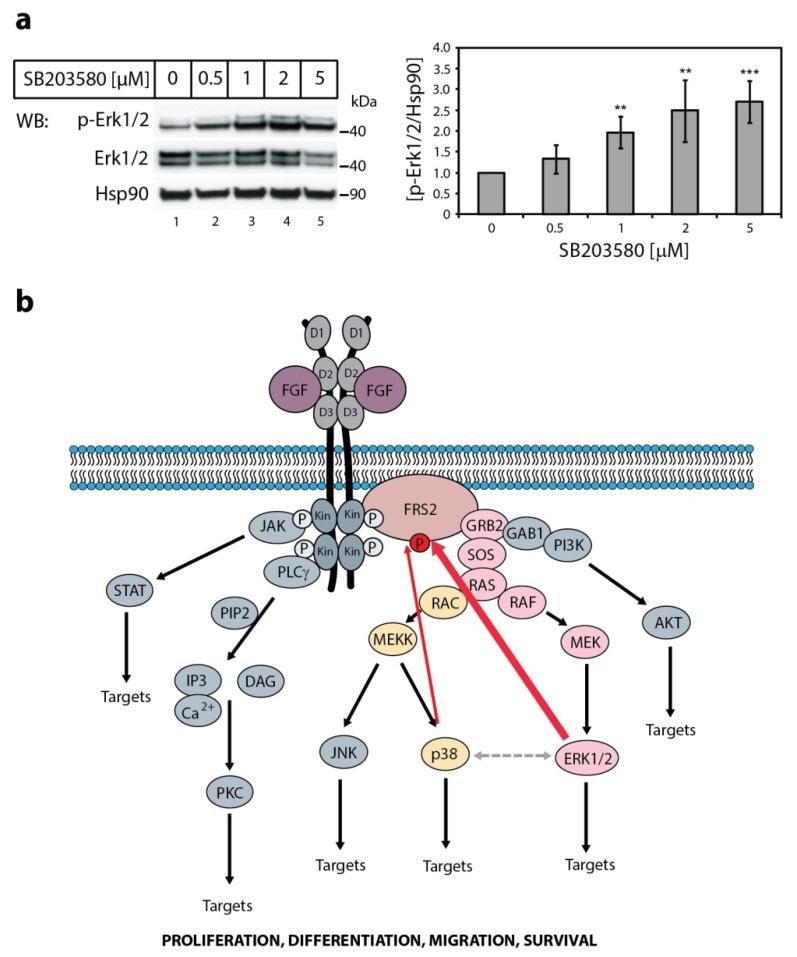
The effect of p38 kinase activity on Erk1/2 activity and FGF1-induced signaling. (**a**) The effect of p38 specific inhibitor on Erk1/2 activity. Serum-starved NIH3T3 cells were treated with increasing concentration of the specific p38 kinase inhibitor SB203580 for 30 min. Then, the cells were lysed and the cellular material was analyzed by SDS-PAGE and immunoblotting using the following antibodies: anti-phospho-Erk1/2 (p-Erk1/2), anti-Erk1/2, and anti-Hsp90 antibodies as a loading control. A representative experiment is shown, n = 4. The graph presents quantification of bands corresponding to phospho-Erk1/2 (p-Erk1/2) normalized to loading control (Hsp90) and expressed as a fold of change in comparison with untreated control. Data are means ± SD of four independent experiments; ** *p* < 0.01, *** *p* < 0.001. (**b**) Schematic representation of synergistic effect of p38 and Erk1/2 in the downregulation of FGF1-induced signaling through FRS2. FGF1-induced tyrosine phosphorylation of FGFR1 leads to the activation of FRS2 followed by GRB2/SOS-mediated activation of RAS and MAP kinases (Erk1/2 and p38). Activated Erks are supported by p38 in phosphorylation of FRS2 (red arrows), constituting a negative feedback loop that results in reduced tyrosine phosphorylation of FRS2 and consequent attenuation of FGFR signaling. Grey dashed line represents functional cross-talk between Erks and p38.

Next, we verified the impact of p38 inhibition on the activity of Erks without FGF1 stimulation. We found that in the presence of increasing concentrations of the specific p38 kinase inhibitor, SB203580, the basal level of phosphorylated Erk1/2 in serum-starved NIH3T3 was augmented (Figure 3a), showing that reduced activity of one MAP kinase (p38) extorted increased activity of the other (Erk1/2). We hypothesize that this crosstalk functions as a backup system in cells. If one of the two kinases is turned off, the other one can take over.

## 3. Discussion

Several mechanisms to attenuate FGFR activity have been reported, however, the overall picture of how the FGFR signaling is controlled has not been yet fully described [6]. FRS2 definitely plays an important role in the regulation of FGFR activity, constituting a key component in the Ras/MAPK pathway and being at the same time under control of its effectors (Erks) in a negative feedback loop [17].

Our results show that p38 kinase phosphorylates FRS2 in vitro, and together with Erk1 and Erk2 takes part in modulation of the duration and the intensity of FGFR signaling. Experiments with anisomycin, a p38 activator, revealed that in specific conditions (e.g., stress), p38 can compensate for Erks’ action. Despite the similar activity in vitro, the effect of p38 on FRS2 phosphorylation in cells is much weaker than the effect of Erk1 and Erk2. It is probably caused by the fact that growth factors, including FGF1, evoke stronger activation of pro-proliferative MAPKs, i.e., Erks, than stress-response MAPKs, such as p38 kinase [20]. We also found that inhibition of p38 slightly increases the basal activity of Erks. It seems that p38 provides a kind of fine-tuning of the Erks-driven feedback system, taking part in the complicated and multilevel network of downregulation mechanisms of FGFR signaling (Figure 3B). However, to elucidate how one MAP kinase can substitute for another in the absence of FGF ligand, further studies are required. We speculate that inhibition of p38 by SB203580, reducing the degree of FRS2 and FGFR basal serine/threonine phosphorylation, evokes minor upregulation of their tyrosine phosphorylation. A slight increase in FGFR and FRS2 activity results in amplification of Erks phosphorylation.

All MAPK family members share a common phosphorylation site motif [22]. Their substrate specificity is achieved through the docking interactions occurring between the regions outside the phosphorylation site in the substrate and fragments distal from the active site in the kinase [22]. The best characterized docking site involved in interaction with MAPK is the D-site, which consists of a basic region, followed by an LXL motif and a hydrophobic region [22,26]. Interestingly, while Erks require three parts of the D-site, p38 can phosphorylate a substrate without the LXL motif [27]. Another conserved motif, known as the DEF site or the FXFP motif, was identified in several substrates of Erks, including transcription factors, phosphatases, scaffolding, and focal adhesion proteins [28]. It was also shown that p38α has a DEF site motif similar to Erk2, being highly selective for aromatic residues at P1 and P3 position with high preference to Trp [22]. Therefore, it is not surprising that Erk1/2 substrate, FRS2, can also be phosphorylated by p38 kinase.

MAPK signaling seems to be a dynamic map of non-linear interactions rather than static connections. Different MAPK cascades operate in parallel, but at the same time they constitute a network and modulate each other’s activity [29]. Here, we have described a cross-talk between two classes of MAPKs in the regulation of FGFR-dependent signaling, but there are other cases of cooperation between different MAPKs. High specificity is observed among upstream kinases, but at the level of MAPK substrate a cross-talk is not unusual [29]. A good example is Elk-1, a member of Ets family of transcription factors controlling the expression of various signaling molecules, which is a target of all three MAPK (Erk1/2, p38, and JNK). Activation of MAPK pathways results in phosphorylation and positive regulation of Elk1, leading to the transcription of early response genes [30]. Another example is MSK1 protein, mitogen-, and stress-activated kinase 1, which is a substrate of both Erks and p38-MAPK during oxidative stress in skeletal myoblasts. Phosphorylation of MSK1 was suggested to influence NF-κB signal specificity in stress response [31]. Another group showed that Erk1/2 and p38 pathways cooperate to promote p21CIP1 expression in order to ensure a sustained G1 cell cycle arrest [32]. The activation of both Erks and p38 was also demonstrated to be essential in regulating delayed STAT3 phosphorylation, as well as in ANF (atrial natriuretic factor) expression profile in response to IL-1β treatment, suggesting their simultaneous role in the development of IL-1β-induced hypertrophy in cardiac myocytes [33]. Furthermore, it was suggested that the function of p38α kinase can be modulated by the cross talk between JNK and p38 kinases [34].

Therapeutic targeting of components of the MAPK cascades is an urgent medical need. Inhibitors of Erks and p38 signaling pathways are in many clinical trials dedicated to treating different types of cancer, inflammation, pain, rheumatoid arthritis, asthma, and neurodegenerative diseases, including Alzheimer’s [35,36]. However, it is under question whether targeting of a single signaling molecule can provide an effective therapy. A growing number of drugs are used in combination. Especially in the case of targeting the Erk pathway, vertical inhibition to block two subsequent steps in the cascade is now standard care in a few types of cancer [36,37]. Moreover, to overcome different compensatory mechanisms generated by signaling feedback loops and cross-talks resulting in resistance, efforts are focused to target multiple pathways simultaneously (horizontal inhibition) by combining selective agents [36,38]. Thus, understanding of interplay between different cascades and signaling components is of great importance. Cross-talk between Erk1/2 and p38 and their potential compensation effect should be taken into account during biochemical studies, and might have implications in the design of effective targeted therapies.

## 4. Materials and Methods

The following primary antibodies were used: rabbit anti-MAPK (Erk1/2, p44/p42; #9102), mouse anti-phospho-MAPK (Erk1/2, p44/p42) (Thr202/Tyr204; #9106), mouse anti-phospho-FGFR (Tyr653/Tyr654; #3476), and rabbit anti-phospho-FRS2α (Tyr196; #3864) from Cell Signaling Technology (Danvers, MA, USA), rabbit anti-FRS2α (H-91; sc-8318), from Santa Cruz Biotechnology (Dallas, TX, USA), mouse anti-phospho-p38 MAPK antibody (Thr180/Tyr182; 612280) and mouse anti-Hsp90 (610418) from BD Transduction Laboratory (San Jose, CA, USA), mouse anti-tubulin (T6557) from Sigma-Aldrich (St Louis, MO, USA). Specific anti-phospho-FGFR1 (Ser777) (pS777-FGFR1) antibody was made by GenScript (Piscataway, NJ, USA) using the following phospho-specific peptide CSMPLDQYpSPSFPDTR. The antibody was purified using the phosphopeptide and by cross-adsorption to the corresponding non-phosphopeptide. HRP-conjugated secondary antibodies were from Jackson Immuno Research Laboratories (West Grove, PA, USA). Heparin-Sepharose CL-6B affinity resin was from GE Healthcare (Piscataway, NJ, USA). Brefeldin A, SB203580, anisomycin, and SL327 were from Calbiochem (San Diego, CA, USA). Heparin, sodium orthovanadate, and U0126 were from Sigma-Aldrich. Phos-tag gels were from Wako Chemicals (Osaka, Japan). All other chemicals were from Sigma-Aldrich.

### 4.1. Cell Lines and Bacterial Strain

NIH3T3 cells were grown in DMEM from Thermo Fisher Scientific (Waltham, MA, USA) or (Biowest, Nuaille, France) supplemented with 10% bovine serum (Thermo Fisher Scientific) and 100 U/mL penicillin and 100 µg/mL streptomycin (Thermo Fisher Scientific). The U2OS cells stably expressing FGFR1 (U2OS-R1) have been described previously [39]. The cells were propagated in DMEM supplemented with 10% fetal bovine serum (Gibco), 100 U/mL penicillin, and 100 µg/mL streptomycin and 0.2 mg/mL geneticin (Invitrogen). For expression of FGF1 wild-type *Escherichia coli* strain Bl21(DE3)pLysS from New England Biolabs (Ipswich, MA, USA) was used.

### 4.2. Recombinant Proteins

GST-tagged FRS2 recombinant protein (Q01) was purchased from Anova (Taipei, Taiwan), active human recombinant p38α MAPK was from R&D Systems (Minneapolis, MN, USA) and active human recombinant MAP kinases, Erk1 (p44) and Erk2 (p42), from Calbiochem. Recombinant FGF1 was produced in *E. coli*, as described previously [40].

### 4.3. Analysis of Signaling Cascades

Serum-starved cells were treated with 20 ng/mL FGF1 in the presence of 10 U/mL heparin and in the presence or absence of indicated inhibitors for 15 min. Signaling kinetics were carried out in the presence or absence of 2 μg/mL brefeldin A. The cells were lysed with SDS sample buffer, scraped, and sonicated. Total cell lysates were separated by SDS-PAGE or by Phos-tag SDS-PAGE, transferred onto Immobilon-P membrane and subjected to immunoblotting. The sectioned membrane was stripped with Restore Western Blot Stripping Buffer (Thermo Fisher Scientific) and re-probed maximally four times with different antibodies. ImageLab software (version 6.0.1) from Bio-Rad (Hercules, CA, USA) was used to quantify the intensity of bands. The intensity of bands corresponding to phospho-proteins (pY-FGFR, pY-FRS2, p-Erk1/2) was normalized to the intensity of bands corresponding to Hsp90 and then expressed as a fraction of control.

### 4.4. In Vitro Phosphorylation of Recombinant FRS2

In vitro phosphorylation experiments were performed with recombinant proteins. One microgram of a fusion protein was incubated with kinases and 40 µCi/mL [γ-33P] ATP in reaction buffer (25 mM HEPES, pH 7.5, 20 mM MgCl2, 1 mM Na2MO3, 20 mM sodium β-glycerophosphate, 1 mM DTT, 5 mM EGTA) at 30 °C for 30 min. The proteins were separated by SDS-PAGE, electroblotted, and analyzed by autoradiography. Equal loading was ensured by membrane staining with Coomassie Blue.

### 4.5. Statistical Analysis

For statistical analysis, one-way analysis of variance (ANOVA) with Tukey’s posttest was applied using SigmaPlot 12 software from (Systat Software, San Jose, CA, USA); *p* < 0.05 was considered statistically significant.

## Abbreviations

FGF	Fibroblast Growth Factor
FGFR	Fibroblast Growth Factor Receptor
FRS2	FGF receptor substrate 2
MAPK	mitogen-activated protein kinase
